# Untargeted Metabolomics Reveals Fruit Secondary Metabolites Alter Bat Nutrient Absorption

**DOI:** 10.1007/s10886-024-01503-z

**Published:** 2024-05-17

**Authors:** Mariana Gelambi, Susan R. Whitehead

**Affiliations:** 1 Department of Biological Sciences, Virginia Polytechnic Institute and State University, Latham Hall RM 427, 220 Ag Quad Lane, Blacksburg, VA 24060 USA; 2 La Selva Biological Station, Organization for Tropical Studies, Puerto Viejo de Sarapiquí, Heredia Province Costa Rica

**Keywords:** *Carollia*, Chemical Ecology, Detoxification, Fecal Metabolome, Frugivores

## Abstract

The ecological interaction between fleshy fruits and frugivores is influenced by diverse mixtures of secondary metabolites that naturally occur in the fruit pulp. Although some fruit secondary metabolites have a primary role in defending the pulp against antagonistic frugivores, these metabolites also potentially affect mutualistic interactions. The physiological impact of these secondary metabolites on mutualistic frugivores remains largely unexplored. Using a mutualistic fruit bat (*Carollia perspicillata*), we showed that ingesting four secondary metabolites commonly found in plant tissues affects bat foraging behavior and induces changes in the fecal metabolome. Our behavioral trials showed that the metabolites tested typically deter bats. Our metabolomic surveys suggest that secondary metabolites alter, either by increasing or decreasing, the absorption of essential macronutrients. These behavioral and physiological effects vary based on the specific identity and concentration of the metabolite tested. Our results also suggest that a portion of the secondary metabolites consumed is excreted by the bat intact or slightly modified. By identifying key shifts in the fecal metabolome of a mutualistic frugivore caused by secondary metabolite consumption, this study improves our understanding of the effects of fruit chemistry on frugivore physiology.

**Supplementary Information:**

The online version contains supplementary material available at 10.1007/s10886-024-01503-z.

## Introduction

During foraging, frugivores encounter a diverse array of secondary metabolites present in fruit pulp (Whitehead et al. 2021). These metabolites encompass a broad range of chemicals that orchestrate ecological interactions by defending the pulp against antagonists and attracting mutualist frugivores (Schoonhoven et al. 2005). Some of these fruit secondary metabolites exhibit wide-ranging bioactivity (Herrera 1982), including toxic (Cipollini and Levey 1997a), anti-nutritional (Gelambi et al. 2024), and deterrent properties (Schaefer et al. 2003; Cazetta et al. 2008; Rojas et al. 2021). While research in the chemical ecology of seed dispersal has predominantly concentrated on investigating the adaptive significance of these fruit metabolites for plants (Whitehead et al. 2021; Nelson et al. 2023), little is known about the broader physiological effects these chemicals exert on mutualistic frugivores.

In Neotropical forests, a ubiquitous seed dispersal interaction involves short-tailed bats (*Carollia*, Phyllostomidae) and their primary food source, pepper plants (*Piper*, Piperaceae), which often contain diverse mixtures of secondary metabolites (Kunz et al. 2011; Uckele et al. 2021; Philbin et al. 2022). In this system, fruit secondary metabolites play a pivotal role in mediating several processes, including bat foraging decisions (Whitehead et al. 2016; Maynard et al. 2020; Hernández-Leal and Sánchez 2021; Gelambi et al. 2024), seed dispersal patterns (Baldwin et al. 2020), and the evolution of the mutualism between fruits and bats (Santana et al. 2021). Although fruit secondary metabolites play a crucial role in shaping these ecological and evolutionary aspects of the *Carollia-Piper* system, the physiological effects that might provide a mechanistic basis for these ecological and evolutionary consequences have not been explored.

Studying the collection of excreted metabolites in feces, known as the fecal metabolome, is a non-invasive initial step in investigating the effects of fruit secondary metabolites on frugivore physiology. Feces contain diverse arrays of metabolites that can serve as direct indicators of the ingestion, processing, and absorption of food (Matysik et al. 2016). Therefore, any alterations in the abundance and composition of excreted metabolites resulting from the consumption of secondary metabolites offer valuable insights into their impact on consumer physiology (Matysik et al. 2016). To investigate these potential alterations, untargeted metabolomics offers a suitable approach, focusing on quantifying and identifying broad classes of small molecules, typically below 1500 Da (Rey-Stolle et al. 2022). Recent untargeted metabolomics studies have captured alterations in the fecal metabolome associated with a range of chemicals and diets across diverse animal taxa, including insects (Qin et al. 2020), fish (Hano et al. 2021), reptiles (Shi et al. 2023), and mammals, such as rats (Vieira-Potter et al. 2018; Torres Santiago et al. 2019) and humans (Jiménez-Girón et al. 2015; Jain et al. 2019). These alterations, often associated with changes in the microbiome, provide insights into the broader effects of specific drugs and chemicals, enabling the identification and quantification of fecal biomarkers linked to certain chemical consumption, and highlighting diet-induced physiological shifts with potential ecological consequences.

Here, we used Seba’s short-tailed fruit bats (*Carollia perspicillata*) to explore the effect of fruit secondary metabolites on bat foraging behavior and digestive physiology. First, we investigated how four secondary metabolites commonly found in plants and reported in *Piper* spp. affect the foraging behavior of captive bats (objective 1). Second, we examined how ingesting these four secondary metabolites alters bat digestive physiology by analyzing changes in the fecal metabolome (objective 2). By addressing these objectives, we improved our understanding of the organismal-level consequences of secondary metabolite consumption for a keystone Neotropical frugivore.

## Methods and Materials

*Study Site and Study Organism.* The study was conducted at La Selva Biological Station, province of Heredia, Costa Rica, between February and March 2021. La Selva, managed by the Organization for Tropical Studies (OTS), encompasses a 1,536-hectare protected lowland area comprising a mix of primary and secondary forests and abandoned plantation areas (McDade et al. 1994). In the study site, short-tailed bats are one of the most abundant groups of fruit bats (*Carollia*, Phyllostomidae). *Carollia* bats are the primary seed dispersers of pepper plants (*Piper*, Piperaceae), a diverse genus of flowering plants (Fleming 2004; Maynard et al. 2019; Santana et al. 2021).

*Secondary Metabolite Selection.* Based on previous phytochemical studies in *Piper* spp. (Salehi et al. 2019) and commercial availability, we selected the alkaloid piperine (285.34 g/mol, Sigma-Aldrich), the polyphenolic tannic acid (1701.20 g/mol, Sigma-Aldrich), the phenolic eugenol (164.20 g/mol, Sigma-Aldrich), and the terpene phytol (296.53 g/mol, Cayman) to test how different classes of secondary metabolites affect the foraging behavior and digestive physiology of bats. We conducted the experiments described below using concentrations of 0.1%, 2%, and 3% of the dry weight of an artificial diet for each metabolite. Similar ranges of concentrations have been reported for these and other secondary metabolites in *Piper* spp. plants (Salehi et al. 2019).

*Bat Capture and Maintenance*. All experiments described below were approved by the Comisión Nacional para la Gestión de la Biodiversidad (resolution number R-007-2021-OT-CONAGEBIO), Virginia Tech Institutional Animal Care and Use Committee protocols (approval no. IACUC 20–212) and Virginia Tech Institutional Biosafety Committee (approval no. 21 − 020) We captured bats using mist nets placed in clearings and secondary forest sites. Upon capture, we released juvenile bats and pregnant females, while adult male and non-reproductive female bats were transferred to individual flight cages (2 × 1 × 1 m) within the forest. We utilized 30 bats for the study, housing them in three groups of ten individuals during three rounds of trials (see below). All bats acclimated to the flight cages the first two nights, where they were fed on a maintenance diet of water, agar powder (Eco-Taste), mashed ripe banana, soy protein isolate powder (Bulk Supplements), NaCl, CaHPO_4_ (Eisen-Golden Laboratories), vegetable oil, and wheat germ (Bob’s Red Mill), using the ingredient proportion suggested in Denslow et al. (1987). After finishing each nightly trial, which lasted approximately three and a half hours in total (see below), bats were fed 35 g of maintenance diet and water *ad libitum*. We cleaned the bottom of the cages daily with a diluted bleach solution (1/10). After finishing the acclimation night and the trials (eight-night total), we released the bats at the capture site. The average percentage change in body mass compared to the initial mass was approximately − 1%.

*Objective 1. The Effects of Secondary Metabolites on the Foraging Behavior of Captive Bats.* To assess the effect of metabolite identity and concentration on *C. perspicillata* preference, we performed non-choice trials to measure the amount of food consumed by each bat within the first 30 min of the evening. Each bat received the four metabolites and two controls (maintenance diet) in a randomized sequence, with either one treatment or control given per night. These trials were repeated with the three groups of bats, and each group received the metabolites in a different concentration in the artificial diet: group 1 received 0.1% of the metabolites, group 2 received 2%, and group 3 received 3% (*N* = 10 bats per treatment/compound concentration). We offered approximately five grams, equivalent to about 0.8 g of dry weight of the experimental diet in a plastic Petri dish at around 7:00 p.m., when bats are actively foraging in their natural habitat. After 30 min, we recorded the weight of the Petri dishes.

*Objective 2. The Effects of Secondary Metabolites Consumption on the Bat Fecal Metabolome.* After recording the amount of food consumed for objective 1, Petri dishes were returned to the flight cages, allowing the bats to consume the remaining diet. Approximately three hours later, we collected the fecal samples resulting from the initial five grams of food offered. To separate feces from urine, we positioned a fine plastic mesh above the cage floor, lined with a plastic sheet, ensuring the fecal samples remained on the mesh while the urine passed through it. We used a clean spatula to collect the fecal samples and stored them in plastic microcentrifuge tubes. Fecal samples were not collected from bats that did not entirely consume the diet. We stored fecal samples at − 80 °C for later analysis in the laboratory, except during transport on dry ice from Costa Rica to Virginia, USA.

*Fecal Metabolome Analysis.* Frozen fecal samples were dried using a Speedvac at 65 °C, 100 mTorr for four hours. As bat fecal samples are potentially contaminated with *Histoplasma capsulatum*, we decontaminated them by adding 1 mL of isopropanol to approximately 10–20 mg of fecal samples. The isopropanol was evaporated using a Speedvac at a temperature of 65 °C, vacuum 100 mTorr for around three hours. Then, we added 10 uL of 1 µg/µL ribitol as the internal standard and 500 µl of 80% methanol. We vortexed the samples for 5 s, sonicated them for 15 min, and then shook them for two hours on an orbital shaker at 140 rpm at room temperature. We centrifuged the samples at 13,000 g for 15 min. We collected 400 µl of the supernatant from each sample into a glass micro insert and evaporated the solvent at 65 °C, vacuum 100 mTorr for one hour. For the derivatization reaction, we added 40 µL of 20 mg/mL methoxyamine hydrochloride in pyridine and incubated for 90 min at 60 °C. Then, we added 40 µL MSTFA (N-methyl-N-(trimethylsilyl)trifluoroacetamide) + 1%TMCS (chlorotrimethylsilane) reagent and incubated for 90 min at 60 °C. Finally, we injected the samples into an Agilent 7820 gas chromatograph paired with a 5977 mass spectrometer equipped with an HP5-MS column (Agilent Technologies, Santa Clara, CA, USA). We converted all Agilent files (.D) to AIA format (.CDF) using ChemStation and processed chromatography data, including peak peaking and alignment, using the R package metaMS (Wehrens et al., 2014). We saved metabolite spectra as .msp files and matched them using the NIST MS search 2.0. In each chromatogram, we removed the peaks detected in the blanks and normalized the instrument response by dividing the peak area of each peak by the peak area of the internal standard and the dry weight of each sample.

*Statistical Analysis.* All the analyses were performed using R version 4.2.1 (R Core Team 2021). To investigate the effects of the four selected secondary metabolites (piperine, tannic acid, eugenol, and phytol) on the foraging behavior of captive bats, we first determined a consumption ratio. This ratio was calculated by dividing the total amount consumed (g) per treatment per individual bat by the average food consumption (g) recorded in the two unsupplemented controls offered to the same bat. The resulting ratio provides insight into the relative preference of each bat for particular secondary metabolites compared to the controls. Ratios greater than one indicate a preference for the treatment over the control, ratios less than one indicate a preference for the control, and ratios of one suggest no preference, implying no deterrent effect of the treatment. Bats not participating in the control trials were excluded from subsequent analyses. We assessed the distribution of the ratios using the ‘shapiro.test()’ function. Given the non-normal distribution of the ratios, we conducted a non-parametric one-sample Wilcoxon test using the function ‘wilcox.test()’, with the mu parameter set to 1, to assess whether the ratios of consumption at a given concentration (0.1, 2, and 3% dry weight) were significantly different from 1.

To examine how secondary metabolite consumption affects the composition of the bat fecal metabolome (objective 2), we used a set of non-metric multidimensional scaling (NMDS) analyses with the Bray-Curtis dissimilarity index. One model was conducted for each concentration and set of trials. To assess the statistical support for differences in composition across compounds, we first examined the homogeneity of group dispersions (PERMDISP2) using the ‘betadisper()’ function, followed by a permutational multivariate analysis of variance (PERMANOVA) using the ‘adonis2()’ function with 999 permutations. To identify the individual excreted metabolites that distinguish the fecal metabolome of bats that ingested different secondary metabolites at various concentrations, we used random forest classification models followed by variable selection using the Boruta algorithm, using the ‘randomForest()’ and ‘Boruta()’ functions respectively. Random forest and Boruta analyses suggest a set of candidate molecules that potentially differ in the fecal metabolome of bats that consumed different secondary metabolites at a given concentration. Then, we tested the direction, i.e., increase or decrease of each excreted metabolite among the treatments, and the statistical support for differences among treatments using a set of generalized linear mixed models (GLMMs) for each excreted metabolite. The GLMMs included each excreted metabolite identified in the Boruta analysis as the response variable, ingested secondary metabolite identity (treatment) as the predictor variable, and bat identity and trial date as random effects in the models coded as random intercepts, i.e., (1|Bat) + (1|Date). Finally, for each excreted metabolite suggested in random forest and Boruta analyses, we obtained the chemical taxonomy classification using the package ‘ClassyFireR’ (Djoumbou Feunang et al. 2016).

Multivariate analyses, including NMDS, PERMANOVA, and multivariate homogeneity of variances (PERMDISP2) were conducted using the ‘vegan’ package (Oksanen et al. 2020). Random forest classification models and variable selection via the Boruta algorithm were performed using the ‘randomForest’ (Liaw et al. 2015) and ‘Boruta’ (Kursa and Rudnicki 2017) packages. The set of GLMMs was constructed using the ‘glmmTMB’ package (Brooks et al. 2017).

## Results

**Fig. 1:**
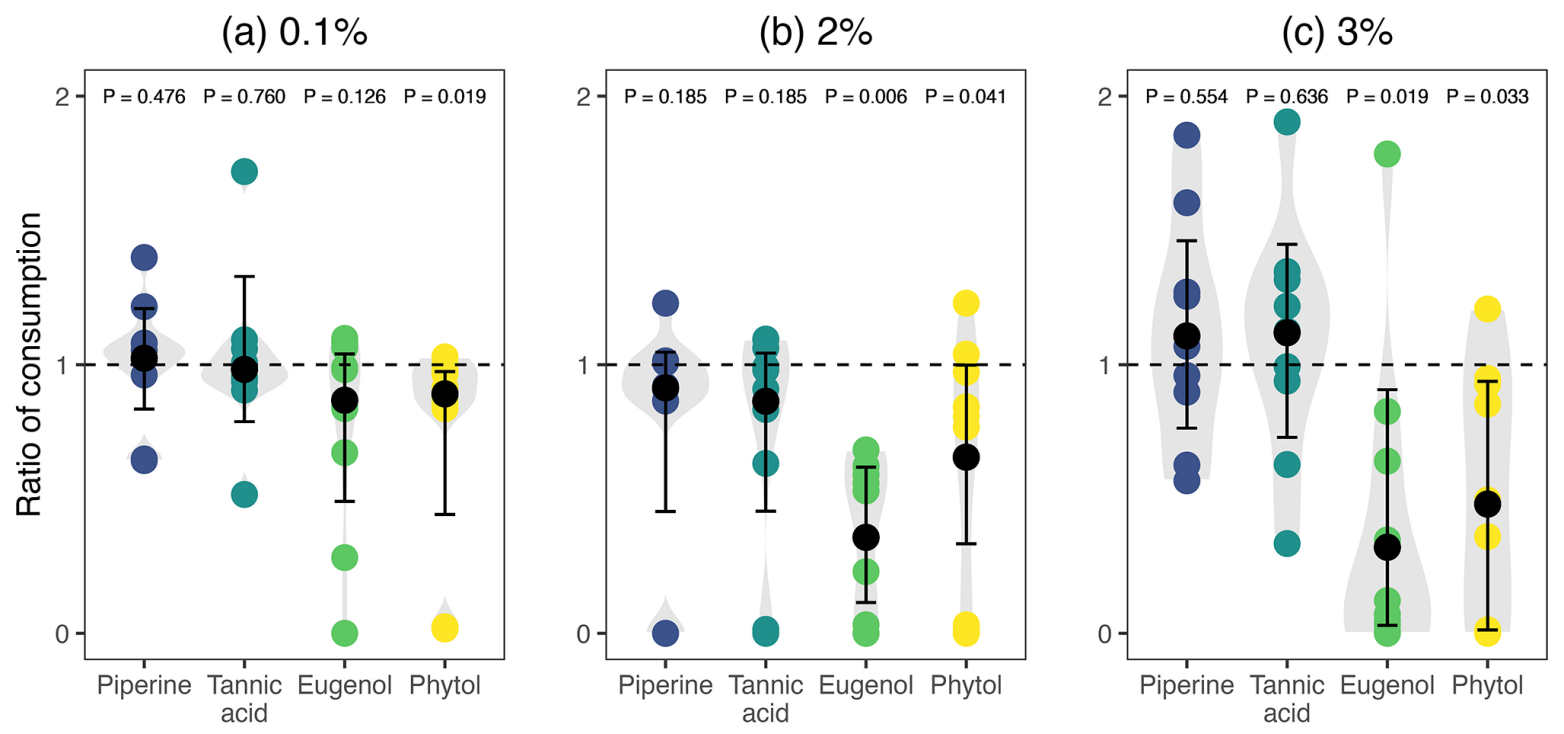
Effect of four secondary metabolites on the ratio of consumption by bats. The ratio was calculated using the total amount of food eaten (g) by each in 30 min divided by the average of control (unsupplemented) food consumed by the same bat. Each data point represents a preference trial, and the shape and color indicate the secondary metabolite identity. Trials were conducted using three different concentrations (a) 0.1, (b) 2, and (c) 3% dry weight. *P*-values were obtained through a one-sample Wilcoxon test, where a significant *P*-value indicates that means are significantly different from 1. Values greater than 1 indicate a preference for the treatment compared to the control, while values lower than 1 indicate a preference for the control. A value of 1 would indicate no preference for either treatment or control and, therefore, no deterrence effect of the metabolite. Black points and error bars represent pseudo-medians and 95% confidence intervals computed using the Wilcoxon test. Comparisons between secondary metabolites tested at a given concentration were conducted using GLMMs (Table S2 and S3)

*Objective 1. The Effects of Secondary Metabolites on the Foraging Behavior of Captive Bats.* The one-sample Wilcoxon test showed that at the three concentrations tested, piperine and tannic acid consumption ratios are not significantly different than 1 (Table S1 and Fig. 1), indicating that these two secondary metabolites do not have a detectable deterrent effect. In contrast, at the three concentrations tested, eugenol and piperine consumption ratios significantly differ from 1 (Table S1 and Fig. 1), suggesting that these two secondary metabolites have an overall deterrent effect. GLMMs (Table S2) post-hoc pairwise comparisons (Table S3) explored the differences in consumption ratios between the four secondary metabolites at a given concentration, indicating that at 2%, piperine and eugenol (*P* < 0.001), as well as tannic acid and eugenol (*P* < 0.001) showed statistically significant differences. Similarly, at 3%, eugenol was significantly different from piperine (*P* < 0.001) and tannic acid (*P* < 0.001), and phytol was significantly different from piperine (*P* = 0.003) and tannic acid (*P* = 0.007).

**Fig. 2:**
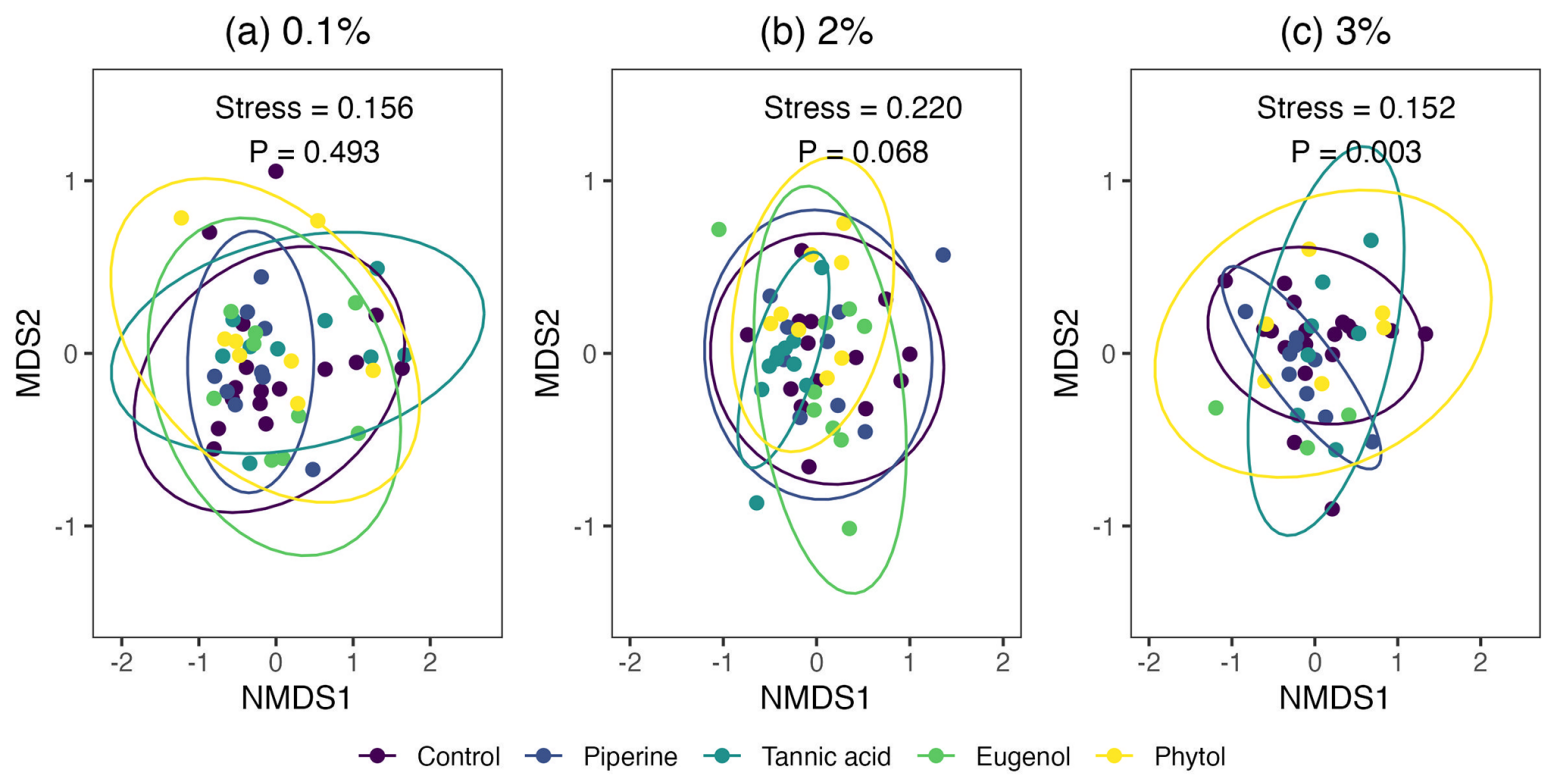
Relatively high concentrations of secondary metabolites affect the composition of the fecal metabolome. Non-metric multidimensional scaling (NMDS) plots show the effect of three different concentrations, (a) 0.1, (b) 2, and (c) 3% dry weight of four secondary metabolites on the fecal metabolome composition. Each point represents a fecal sample colored by the secondary metabolites ingested. Ellipses represent 95% confidence intervals around group centroids. Due to the limited number of samples, ellipses could not be calculated for 3% (C) eugenol

*Objective 2. The Effects of Secondary Metabolites Consumption on the Bat Fecal Metabolome.* At 0.1%, the NMDS analyses did not detect statistically significant clustering among treatment groups (Table S4 and Fig. 2). Conversely, at the highest concentrations tested, 2 and 3%, the ingestion of different secondary metabolites produced significant clustering (Table S4 and Fig. 2), indicating potential shifts in the composition of the pool of excreted metabolites caused by the specific secondary metabolites initially consumed. In none of the groups evaluated was evidence of differences in dispersion across groups (Table S5).

**Fig. 3:**
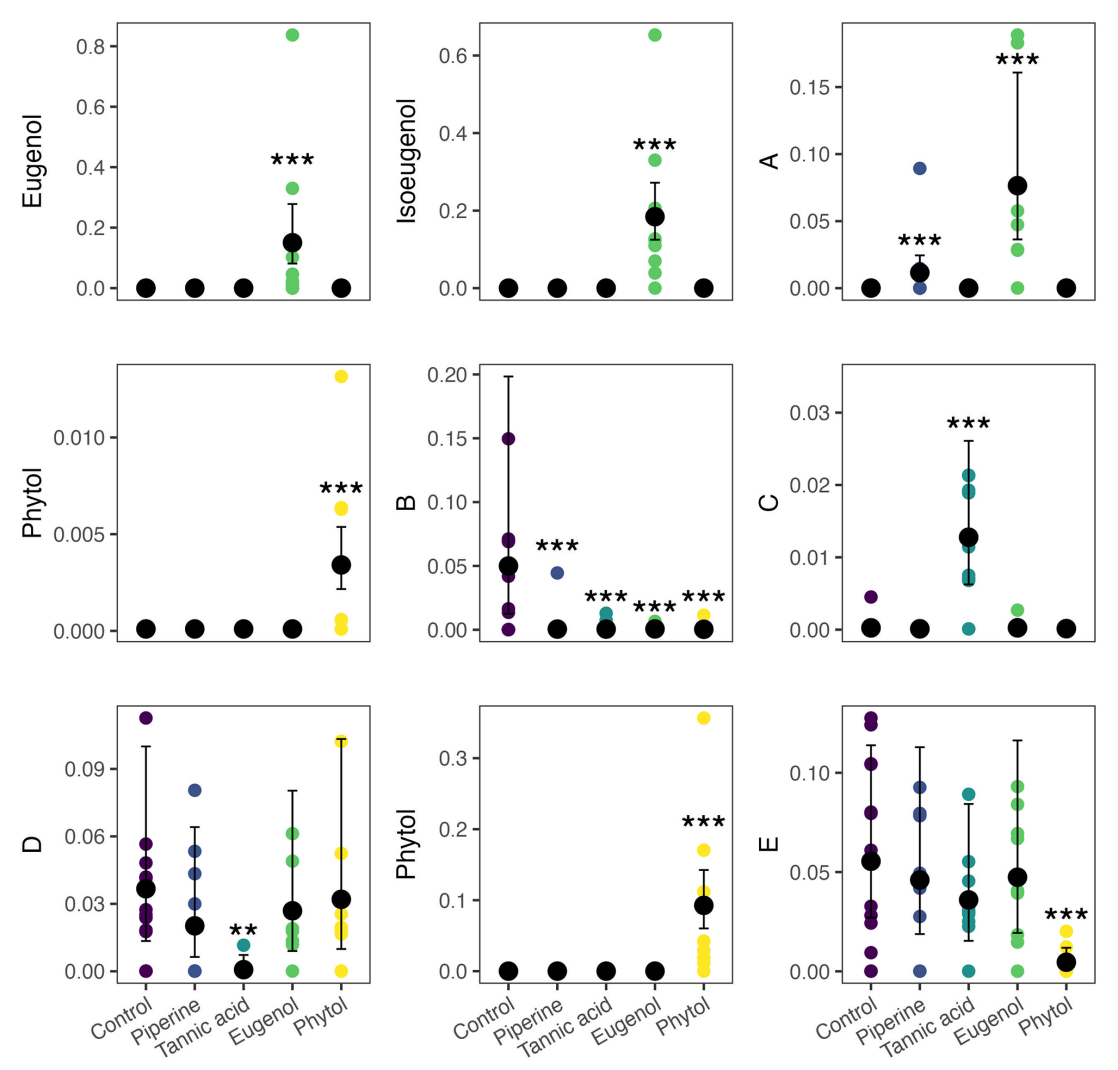
Metabolites identified in the random forest and Boruta analyses between the fecal metabolome of bats that ingested four secondary metabolites (piperine, tannic acid, eugenol, and phytol) at 2%. The effect of metabolite identity was calculated using GLMMs. Parameters predicted by the GLMMs are summarized in Table S6. Best match in the NIST library, IUPAC names: **A**: trimethylsilyl 3-methyl-2-(trimethylsilylamino)-3-trimethylsilylsulfanylbutanoate; **B**: diethoxy-methyl-octadecylsilane; **C**: *N*-(2,6-diethylphenyl)-1,1,1-trifluoromethanesulfonamide; **D**: trimethylsilyl 2-oxo-3-trimethylsilylpropanoate; **E**: trimethylsilyl (*Z*)-octadec-9-enoate. Significance levels are denoted by asterisks, (*P* < 0.001 = ‘***’, *P* < 0.01 = ‘**’, *P* < 0.05 = ‘*’), compared to the control

Then, we investigated the individual excreted metabolites among the two significant treatments in the NMDS, i.e., 2 and 3%. At 2%, the random forest and Boruta analyses suggested nine individual features (Table S6 and S7 and Fig. 3), two of which matched with intact eugenol. Two signals for the same compound (e.g., phytol, eugenol) are likely due to heterogeneity during the derivatization reaction, a common phenomenon in metabolomics where a single metabolite can show numerous signals or features (Deda et al. 2019). At this concentration, eugenol consumption led to the excretion of intact eugenol, isoeugenol, and increased excretion of amino acids, peptides, and analogues, compared with the control. Tannic acid consumption led to an increased excretion of the compound N-(2,6-diethylphenyl)-1,1,1-trifluoromethanesulfonamide, and reduced excretion of a Carboxylic acids and derivatives. Phytol consumption led to the excretion of intact phytol, and reduced excretion of a fatty acid.

**Fig. 4:**
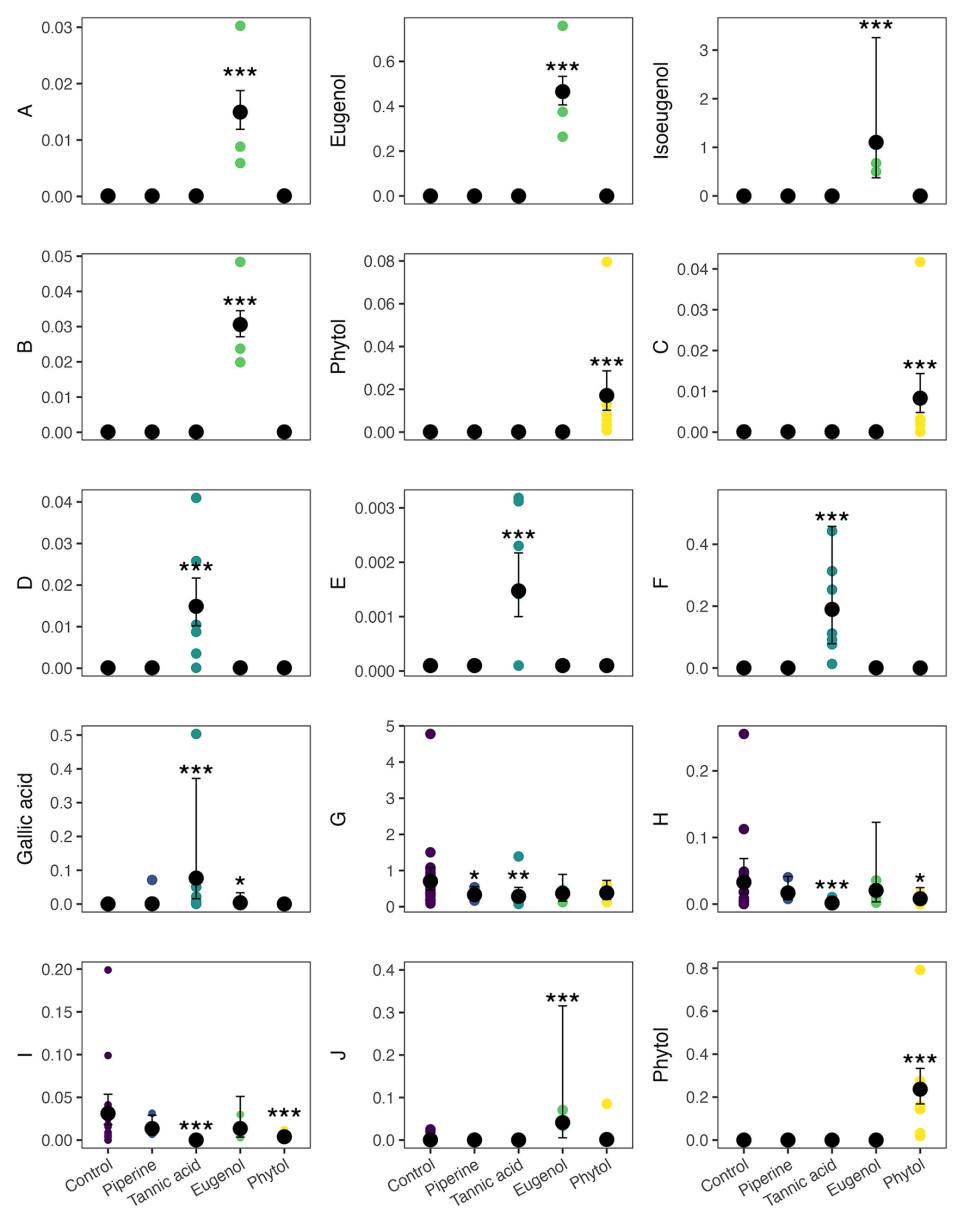
Metabolites identified in the random forest and Boruta analyses between the fecal metabolome of bats that ingested four secondary metabolites (piperine, tannic acid, eugenol, and phytol) at 3%. The effect of metabolite identity was calculated using GLMMs. Parameters predicted by the GLMMs are summarized in Table S6. Best match in the NIST library, IUPAC names: **A**: trimethyl(trimethylsilyloxy)silane; **B**: trimethyl-[(3*Z*)-9-trimethylsilyloxyundeca-3,10-dien-6-yn-5-yl]oxysilane; **C**: hexadec-1-yne; **D**: (*NE*)-*N*-[1-(2,5-dimethoxyphenyl)propan-2-ylidene]hydroxylamine; **E**: 6-[6-amino-8-(2-aminoethylamino)purin-9-yl]-2-hydroxy-2-oxo-4a,6,7,7a-tetrahydro-4 H-furo[3,2-d][1,3,2]dioxaphosphinin-7-ol; **F**: *N*-(2,6-diethylphenyl)-1,1,1-trifluoromethanesulfonamide; **G**: trimethylsilyl hexadecanoate; **H**: methyl (9*E*,15*E*)-octadeca-9,15-dienoate; **I**: methyl (*E*)-dodec-9-enoate; **J**: tert-butyl-hexadecoxy-dimethylsilane. Significance levels are denoted by asterisks, (*P* < 0.001 = ‘***’, *P* < 0.01 = ‘**’, *P* < 0.05 = ‘*’), compared to the control

**Fig. 5:**
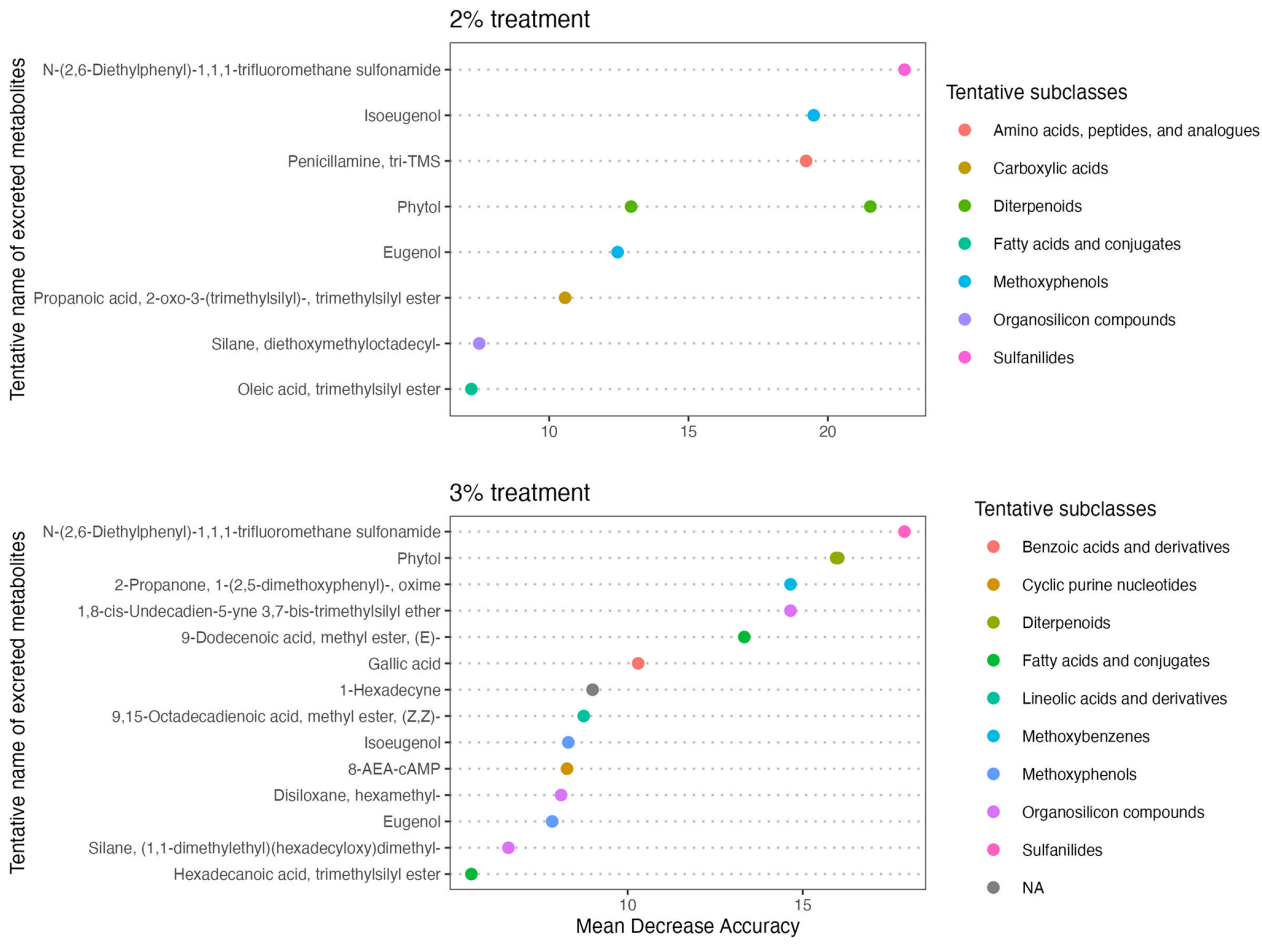
Tentative subclasses of excreted metabolites identified variables distinguishing the fecal metabolome between treatments (piperine, tannic acid, eugenol, and phytol) at 2% and 3% concentrations. Mean Decrease Accuracy is a metric of the importance of each variable in classifying data, showing the reduction in model accuracy when excluding each variable. Higher values indicate more significant importance for accurate classification

At 3%, the random forest and Boruta analyses suggested 15 individual features (Table S6 and S7 and Fig. 4). At this concentration, eugenol consumption led to the excretion of intact eugenol, isoeugenol, and the increased excretion of organosilicon compounds. Tannic acid consumption led to an increased excretion of the compound N-(2,6-diethylphenyl)-1,1,1-trifluoromethanesulfonamide, and increased excretion of methoxybenzenes, nucleotides, sulfanilides, and decrease the excretion of fatty acids. Phytol consumption led to the excretion of intact phytol, increased the excretion of sesquiterpenoids, and decreased excretion of fatty acids, pyrimidines, and pyrimidine derivatives. All secondary metabolites led to the excretion of an imidazolidine (Fig. 5).

## Discussion

Secondary metabolites are abundant and diverse in fruit tissues (Whitehead et al. 2021), yet their effect on frugivore physiology has been largely unexplored. In this study, we investigated the impact of four representative secondary metabolites on the behavior and digestive physiology of fruit bats. Our findings from the behavioral trials revealed that secondary metabolites generally decreased food intake, with the level of deterrence contingent upon the metabolite concentration. After consumption, our results showed that the four secondary metabolites tested altered the pool of metabolites excreted by bats. Each tested metabolite had a distinct impact on modifying the fecal metabolome, presumably due to its varying chemical properties, such as polarity and size. Among the four metabolites tested, tannic acid led to the highest number of changes in metabolite excretion, altering the absorption of key nutrients, followed by eugenol and phytol, which also modified nutrient absorption. Piperine did not lead to significant changes in the fecal metabolome. Furthermore, our findings indicate that a fraction of the studied metabolites, except for piperine, are excreted partially intact or intact.

Our results from the behavioral trials showed that bats are deterred by the secondary metabolites tested. From the perspective of animals, reducing the amount of food consumed per unit of time can be an effective strategy to avoid the accumulation of potentially harmful concentrations of secondary metabolites. From the plant perspective, our results are consistent with the trade-off hypothesis (Cipollini and Levey 1997b), which posits that secondary metabolites that protect the fruit against pathogens also deter mutualists, such as effective seed dispersers. We did not find any pattern that suggests that the most deterrent metabolite causes the most negative effect after consumption in physiology, yet the number of metabolites tested in our study was limited. However, any food aversion due to negative consequences of metabolite consumption could not be evidenced in our study because we offered each metabolite to each bat once. Similarly, other studies have not identified an association between the deterrent effects of plant metabolites and the physiological effects associated with the consumption of the metabolites (Pass and Foley 2000). In sum, the deterrent effect of the four metabolites tested in this study is likely driven primarily by negative stimuli of the olfactory and taste receptors during diet consumption, rather than a post-consumption negative effect, and might represent a strategy to avoid the accumulation of metabolites in the body. However, we anticipate that bats could consume high concentrations of secondary metabolites in certain situations, possibly influenced by seasonality, reproductive status, and health. For example, fruit bats might ignore the deterrent effect of some metabolites to increase nutrient intake (Gelambi et al. 2024). Additionally, as reported in other species (Raman and Kandula 2008), bats might even use plant metabolites for self-medication.

Our untargeted metabolomic survey of the fecal samples reveals that the identity of the ingested secondary metabolites significantly affects the pool of metabolites excreted by bats at 2 and 3%. The random forest and Boruta analyses identified a set of candidate metabolites that potentially drive the clustering patterns found in the NMDS. Tannic acid, and tannins in general, are widely known in nutritional ecology for impeding protein absorption (Barbehenn & Constabel, 2011). Our results suggest that tannic acid can also modify lipid metabolism. Bats excreted significantly fewer fatty acids, suggesting that tannic acid might increase the absorption of fatty acids. Compared to the control, the consumption of the lowest (0.1%) concentration of tannic acid also reduced the excretion of amino acids, peptides, and analogues. Taken together, our results suggest that the consumption of tannic acid significantly affects the absorption of main nutrients. Compared to tannic acid, eugenol and phytol are less studied in the nutritional ecology literature. In our study, eugenol significantly modified the excretion of several metabolites, both increasing and decreasing their excretion. Phytol mainly affects the excretion of lipids and lipid-like molecules, decreasing the excretion of fatty acids. Finally, piperine did not show any significant changes except for the excretion of a nitrogen-containing molecule, which is likely the product of metabolized piperine. Overall, the potential increased lipid absorption reported when consuming diets supplemented with 3% of some of the metabolites could be a strategy to maximize energy obtaining and fuel potential detoxification mechanisms. Additionally, it has been shown that there is an intimate relationship between the pathways involved in the dietary chemical detoxification of enzymes, mainly cytochromes P450, and lipid metabolism (Finn et al. 2009).

One key finding of our study was the detection of intact metabolites in the fecal samples. We also detected signals of intact eugenol, phytol, and gallic acid, which are the subunits of tannic acid, in the fecal samples. Intact piperine was not detected in our samples. In addition to the excretion of intact secondary metabolites, we also detect structurally similar metabolites that are potentially the metabolized form of the initial metabolites.

Excretion of intact or slightly modified fruit secondary metabolites might benefit bats and plants, fueling the mutualisms between the two groups. From the animal perspective, mammalian detoxification pathways are energetically expensive (Sorensen et al. 2005; Au et al. 2013), therefore excreting intact or partially intact secondary metabolites likely represents a mechanism to save energy while consuming fruit pulp rich in secondary metabolites. This is relevant for our focal species, *Carollia perspicillata*, which encounters a diverse and complex array of secondary metabolites in ripe *Piper* pulp. The direct excretion of secondary metabolites has been reported for herbivores, including insects (Cooper 2001; Wang et al. 2021) and mammals (Huang et al. 2016). More specifically for the metabolites studied here, studies conducted in rats show eugenol and piperine are primarily metabolized in sulfate, glucuronic acid, and glutathione conjugated forms (Ganesh Bhat and Chandrasekhara 1986; Thompson et al. 1991; Suresh and Srinivasan 2010). However, detoxification pathways in fruit bats have not been studied, and other strategies (e.g., Voigt et al. 2008) might play an important role in their ability to cope with plant secondary metabolites. Finally, understanding how bats cope with natural plant metabolites can provide the groundwork to explore the effect of anthropogenic toxins, such as pesticides, on bat physiology (Torquetti et al. 2021). Untargeted metabolomics of fecal samples are promising tools that can be used to assess the effects of anthropogenic toxins on bats.

From the plant perspective, after being excreted by bats, intact fruit secondary metabolites in fecal samples might continue functioning as chemical defenses. For instance, the major metabolites identified in bat samples associated with the ingested secondary metabolites, namely, eugenol (Marchese et al. 2017), phytol (Islam et al. 2018), and gallic acid (Choubey et al. 2018), show antimicrobial properties against fungi and bacteria. As pathogen growth decreases the seed emergence success of some plant species (Gallery et al. 2010), we speculate that secondary metabolites might decrease or inhibit the growth of pathogens in the digested pulp containing the seeds. Also, bat fecal samples are occasionally a mix of seeds from different plant species. Although the role of seed interspecific competition in the seed dispersal process has not been studied, secondary metabolites in mixed seed fecal samples might negatively affect the germination of the competitor seeds (Loiselle 1990). As seeds would be dispersed together in one event, the inhibition of germination of seeds from different species might be a mechanism of avoidance of competition for resources.

A limitation in our study that could restrict its generalizability is the use of commercially available secondary metabolites. Although our findings illustrate the distinct impact of each metabolite on the excreted pools of metabolites by bats, it is plausible that in natural conditions, where frugivores consume complex mixtures of secondary metabolites, the physiological effects are more pronounced than the ones reported here. Future studies could explore the likely synergistic effects of secondary metabolite mixtures on frugivore physiology.

In conclusion, our results suggest that secondary metabolites can affect multiple aspects of the physiology and behavior of bats. We reported a set of physiological consequences associated with ingesting secondary metabolites, primarily linked to the alteration of key nutrient absorption. Our experiments also revealed two main strategies that fruit bats might use to cope with fruit secondary metabolites: reducing food consumption per unit of time and excreting intact or slightly modified fruit metabolites. We hypothesize that the excretion of intact defensive fruit metabolites could benefit bats by reducing energy expenditure in their detoxification pathways. Our results have improved our understanding of the organismal-level physiological consequences of ingesting secondary metabolites.

## Electronic Supplementary Material

Below is the link to the electronic supplementary material.

Supplementary Material 1

Supplementary Material 2

## Data Availability

All data, metadata, and R scripts used to generate results and figures (Gelambi & Whitehead, 2024) are available from Zenodo DOI 10.5281/zenodo.11002932. No novel code was generated in this study. The study relies on pre-existing code cited in the Method section.
