# Supplementary material for: Untargeted Metabolomics Reveals Fruit Secondary Metabolites Alter Bat Nutrient Absorption
Source: J Chem Ecol. 2024 May 17;50(7-8):385–96. doi: 10.1007/s10886-024-01503-z (PMC11399193; doi:10.1007/s10886-024-01503-z)
Supplement: Supplementary file 2 — Supplementary Material 2 [file 10886_2024_1503_MOESM2_ESM.docx]

Supplementary information

Untargeted Metabolomics Reveals Fruit Secondary Metabolites Alter Bat Nutrient Absorption

MARIANA GELAMBI^1,2^* AND SUSAN R. WHITEHEAD^1,2^

^1^Department of Biological Sciences, Virginia Polytechnic Institute and State University, Latham Hall RM 427, 220 Ag Quad Lane, Blacksburg, VA 24060. ^2^La Selva Biological Station, Organization for Tropical Studies, Puerto Viejo de Sarapiquí, Heredia Province, Costa Rica

*Corresponding author: Mariana Gelambi, gelambi@vt.edu, 0000-0002-6443-5420

*Objective 1. The effects of secondary metabolites on the foraging behavior of captive bats*

**Table S1** One-sample Wilcoxon signed−rank test with continuity correction for consumption ratios of four secondary metabolites at three concentrations (0.1, 2, and 3% dw), (P < 0.001 = ‘***’, P < 0.01 = ‘**’, P < 0.05 = ‘*’)

| Group | n | V | CI 95% | Pseudo-median | P-value |
| --- | --- | --- | --- | --- | --- |
| 0.1% | | | | | |
| Piperine | 10 | 35 | (0.834, 1.209) | 1.024 | 0.476 |
| Tannic acid | 10 | 24 | (0.788, 1.329) | 0.982 | 0.76 |
| Eugenol | 10 | 12 | (0.491, 1.040) | 0.868 | 0.126 |
| Phytol | 10 | 4 | (0.442, 0.975) | 0.891 | 0.019 |
| 2% | | | | | |
| Piperine | 10 | 14 | (0.454, 1.047) | 0.914 | 0.185 |
| Tannic acid | 10 | 14 | (0.454, 1.043) | 0.864 | 0.185 |
| Eugenol | 10 | 0 | (0.115, 0.618) | 0.357 | 0.006*** |
| Phytol | 10 | 7 | (0.334, 0.998) | 0.656 | 0.041* |
| 3% | | | | | |
| Piperine | 9 | 28 | (0.764, 1.462) | 1.107 | 0.554 |
| Tannic acid | 9 | 27 | (0.730, 1.448) | 1.119 | 0.636 |
| Eugenol | 10 | 4 | (0.030, 0.907) | 0.32 | 0.019** |
| Phytol | 9 | 4 | (0.013, 0.938) | 0.481 | 0.033* |

**Table S2** Generalized linear mixed model (GLMM) exploring the effect of four secondary metabolites (piperine, eugenol, tannic acid, and phytol) at three different concentrations (0.1, 2, and 3% dw). Date was included as random effects in the model. (P < 0.001 = ‘***’, P < 0.01 = ‘**’, P < 0.05 = ‘*’)

| Metabolite | Estimate | SE | CI 95% | z | P-value |
| --- | --- | --- | --- | --- | --- |
| 0.1% | | | | | |
| Piperine (intercept) | 1.017 | 0.108 | (0.80, 1.23) | 9.405 | <0.001*** |
| Tannic acid | 0.011 | 0.110 | (−0.20, 0.23) | 0.097 | 0.923 |
| Eugenol | −0.213 | 0.096 | (−0.40, −0.03) | −2.231 | 0.026** |
| Phytol | −0.238 | 0.099 | (−0.43, −0.04) | −2.407 | 0.016** |
| 2% | | | | | |
| Piperine (intercept) | 0.778 | 0.122 | (0.54, 1.02) | 6.384 | <0.001*** |
| Tannic acid | −0.018 | 0.079 | (−0.17, 0.14) | −0.234 | 0.815 |
| Eugenol | −0.392 | 0.081 | (−0.55, −0.23) | −4.855 | <0.001*** |
| Phytol | −0.137 | 0.080 | (−0.29, 0.02) | −1.714 | 0.087 |
| 3% | | | | | |
| Piperine (intercept) | 1.18017 | 0.15237 | (0.88, 1.48) | 7.746 | <0.001*** |
| Tannic acid | −0.05943 | 0.15999 | (−0.37, 0.25) | −0.371 | 0.710 |
| Eugenol | −0.79265 | 0.15471 | (−1.10, −0.49) | −5.123 | <0.001*** |
| Phytol | −0.61379 | 0.15999 | (−0.93, −0.30) | −3.836 | <0.001*** |

**Table S3** Post−hoc pairwise comparisons using estimated marginal means exploring the effect of four secondary metabolites (piperine, eugenol, tannic acid, and phytol) at three concentrations (0.1, 2, and 3% dw). P-values were adjusted using the Tukey method for comparing a family of four estimates. (P < 0.001 = ‘***’, P < 0.01 = ‘**’, P < 0.05 = ‘*’)

| Contrast | Estimate | SE | df | t−ratio | P-value |
| --- | --- | --- | --- | --- | --- |
| 0.1% | | | | | |
| Piperine - Tannin acid | −0.011 | 0.110 | 33 | −0.097 | 1.000 |
| Piperine - Eugenol | 0.213 | 0.096 | 33 | 2.231 | 0.136 |
| Piperine - Phytol | 0.238 | 0.099 | 33 | 2.407 | 0.095 |
| Tannin acid - Eugenol | 0.224 | 0.111 | 33 | 2.024 | 0.200 |
| Tannin acid -Phytol | 0.248 | 0.105 | 33 | 2.359 | 0.105 |
| Eugenol - Phytol | 0.025 | 0.098 | 33 | 0.249 | 0.994 |
| 2% | | | | | |
| Piperine - Tannin acid | 0.018 | 0.079 | 33 | 0.234 | 0.995 |
| Piperine - Eugenol | 0.392 | 0.081 | 33 | 4.855 | <0.001*** |
| Piperine - Phytol | 0.137 | 0.080 | 33 | 1.714 | 0.333 |
| Tannin acid - Eugenol | 0.374 | 0.082 | 33 | 4.560 | <0.001*** |
| Tannin acid - Phytol | 0.118 | 0.080 | 33 | 1.472 | 0.465 |
| Eugenol - Phytol | −0.255 | 0.080 | 33 | −3.187 | 0.016 |
| 3% | | | | | |
| Piperine - Tannin acid | 0.059 | 0.160 | 30 | 0.371 | 0.982 |
| Piperine - Eugenol | 0.793 | 0.155 | 30 | 5.123 | <0.001*** |
| Piperine - Phytol | 0.614 | 0.160 | 30 | 3.836 | 0.003** |
| Tannin acid - Eugenol | 0.733 | 0.155 | 30 | 4.735 | <0.001*** |
| Tannin acid -Phytol | 0.554 | 0.157 | 30 | 3.523 | 0.007** |
| Eugenol - Phytol | −0.179 | 0.155 | 30 | −1.155 | 0.659 |

*Objective 2. The effects of secondary metabolites consumption on the bat fecal metabolome*

**Table S4** Permutational multivariate analysis of variance (PERMANOVA) evaluating the effect of four secondary metabolites consumption on the fecal metabolome composition. (P < 0.001 = ‘***’, P < 0.01 = ‘**’, P < 0.05 = ‘*’)

| Group | Df | Sum of squares | R2 | Pseudo-F | P-value |
| --- | --- | --- | --- | --- | --- |
| 0.1% | 4 | 0.864 | 0.077 | 0.978 | 0.493 |
| 2% | 4 | 0.997 | 0.111 | 1.408 | 0.068 |
| 3% | 4 | 1.266 | 0.169 | 1.929 | 0.003** |

**Table S5** ANOVA of multivariate homogeneity of group dispersions evaluating the effect of four secondary metabolites consumption on the fecal metabolome composition

| Group | Df | Sum of squares | Mean | F | P-value |
| --- | --- | --- | --- | --- | --- |
| 0.1% | 4 | 0.027 | 0.007 | 0.662 | 0.621 |
| 2% | 4 | 0.057 | 0.014 | 1.388 | 0.253 |
| 3% | 4 | 0.065 | 0.0163 | 1.696 | 0.171 |
